# Can long-read sequencing tackle the barriers, which the next-generation could not? A review

**DOI:** 10.3389/pore.2024.1611676

**Published:** 2024-05-16

**Authors:** Nikolett Szakállas, Barbara K. Barták, Gábor Valcz, Zsófia B. Nagy, István Takács, Béla Molnár

**Affiliations:** ^1^ Department of Biological Physics, Faculty of Science, Eötvös Loránd University, Budapest, Hungary; ^2^ Department of Internal Medicine and Oncology, Faculty of Medicine, Semmelweis University, Budapest, Hungary; ^3^ HUN-REN-SU Translational Extracellular Vesicle Research Group, Budapest, Hungary

**Keywords:** sequencing, short-read, long-read, bioinformatics, DNA

## Abstract

The large-scale heterogeneity of genetic diseases necessitated the deeper examination of nucleotide sequence alterations enhancing the discovery of new targeted drug attack points. The appearance of new sequencing techniques was essential to get more interpretable genomic data. In contrast to the previous short-reads, longer lengths can provide a better insight into the potential health threatening genetic abnormalities. Long-reads offer more accurate variant identification and genome assembly methods, indicating advances in nucleotide deflect-related studies. In this review, we introduce the historical background of sequencing technologies and show their benefits and limits, as well. Furthermore, we highlight the differences between short- and long-read approaches, including their unique advances and difficulties in methodologies and evaluation. Additionally, we provide a detailed description of the corresponding bioinformatics and the current applications.

## Introduction

The complete genetic information of the organisms is stored and transferred in single- and double-stranded ribonucleic (RNA) and deoxyribonucleic (DNA) acids [1]. The mystery behind rare genetic conditions, like chromosomal irregularities or unique sequence variation and mutation profiles in cancer, induced the need of molecular examination at deeper levels. For many years, only a deficient tool set was available to get a better insight into the genetic attributes of genomes. This encouraged the development of novel technologies, such as RNA and DNA sequencing methods. Provoked by the technical and computational progress of the past 50 years, the features of sequence determination changed and evolved. In the early periods, only a few hundred bases were reachable in length; however, the emergence of long-read technologies allowed the reading of longer genomic sequences even with thousands of kilo bases.

The timeline of the sequencing techniques’ evolution can be divided into three main parts: first-generation (FGS), next-generation (NGS), and third-generation (TGS) sequencing. Before short-read NGS approaches became available, FGS techniques were the only tools capable of describing the nucleic acid sequence of different organisms. Thus, their main advantage is that they emphasized the need to use and develop novel sequencing methods to get a deeper knowledge regarding DNA and RNA sequences with repetitive regions, alternative bases, splicing variants and telomeric regions. Later in time, the NGS and mainly TGS methods were capable of opening closed doors for the detection of the listed alteration types, thereby exploring many reasons (and also the curing solution) for diseases. In our review, we strived to show FGS techniques from this point of view, without explaining their applications and attributes in more detail. In this scope, FGS were the pioneers of sequencing around the 1980s, including Sanger’s chromatography and Maxam-Gilbert’s chemical modification-based assays. In the early times, these technologies allowed focusing on relatively small genomes with a few hundred base pairs (bp) in length [2]. Sanger’s idea was to sequence the DNA strand by chain termination. Consequently, in this case, the DNA fragments were converted into chains by DNA polymerases and by the incorporation of nucleotides [3]. Maxam and Gilbert provided a process, during which the sequences of DNA fragments were determined using the combination of radiolabeling, chemical cleaving, and gel electrophoresis of nucleotides, and autoradiography served as the detection method [4].

The second generation, namely, NGS, includes pyrosequencing [5] and sequencing-by-synthesis [6] approaches. They have a feature in common, which is that DNA polymerase moves along the template DNA and sequencing is performed by catalyzing the incorporation of deoxynucleotide triphosphates (dNTPs) in a new complementary DNA strand [7]. Pyrosequencing is a sequence-based form, where a pyrophosphate is released, when dNTPs are sequentially added to the end of a nascent DNA fragment [8]. Sequencing-by-synthesis is the construction of a nucleic acid chain from the emission spectra of fluorescently labeled nucleotides [6].

Although NGS provides more acceptable error rates and more sophisticated sequencing results than FGS, they have several weaknesses that should be mentioned. Read lengths are shorter than demanded, that is why they are referred to as short read techniques nowadays. Consequently, their shortness limits the study of full-length transcript variants, centromere and telomere genomic regions, and gene fusions [9]. Additionally, they are unable to resolve repetitive regions of the genome, making genetic variations challenging to identify, including repeat expansion disorders and structural variants [10]. Extreme guanine-cytosine (GC) content or sequences with multiple homologous elements in the genome and the epigenetically modified bases of DNA and RNA, like N6-methyladenosine (6mA), 5-methylcytosine (5mC), and 5-hydroxymethylcytosine (5hmC) are challenging to characterize with NGS [11]. PCR amplification is essential, which results in higher costs and longer times in the overall sequencing and evaluation process, involves the usage of large equipment and laborious experimental procedures, and expands the bioinformatics analysis with a data preprocessing step. To overcome the limitations, further sequencing techniques have been developed, the representatives of the TGS family, often referred to as long-read sequencing methods [12, 13].

Many scientific papers describing the methodology, evaluation and use of different sequencing assays become available yearly. Currently, long-read TGS and short-read NGS methods are used problem-specifically, either interchangeably or in combination. Although both methods have their own advantages and disadvantages, reviews setting against the methods cannot be found among the currently available publications. Encouraged by this, our goal is to provide a general comparison of long- and short-read techniques. In the present paper, we aimed to review the development of sequencing assays, presenting a brief characteristics of FGS, NGS and TGS, with special emphasis on the possibilities offered by the TGS methods. We also detail the bioinformatics approaches along with the aspects considered during evaluation, as well as related clinical and biological applications.

## Long-read sequencing

TGS provides more precise mapping of reads for reference genomes, promotes different variant detection methods, and offers new solutions for characterizing the epigenetic diversity [14]. In contrast to NGS systems, the generated data is analyzed in real-time and generally, PCR amplification steps are not required before sequencing due to natural isolated nucleic acid strands can be read as well. The longer sequenced reads are the consequences of the improved sequencing chemistries [15, 16]. The increased sequencing speed and accuracy during experiments and the higher quality bioinformatics results also mark the effectiveness of the newly emerged technologies and the inherent chemical kits [17].

TGS technologies conceal the opportunity to emerge as long-term applicable tools in the future. As they provide the long-read sequencing of whole genomes, their usage in the field of genomics entails the chance of more and more accurate description of both human and non-human genetic diversities. Furthermore, improvements aimed to decrease costs and analysis time could invoke their application in routine diagnostics.

### Nanopore sequencing

**FIGURE 1:**
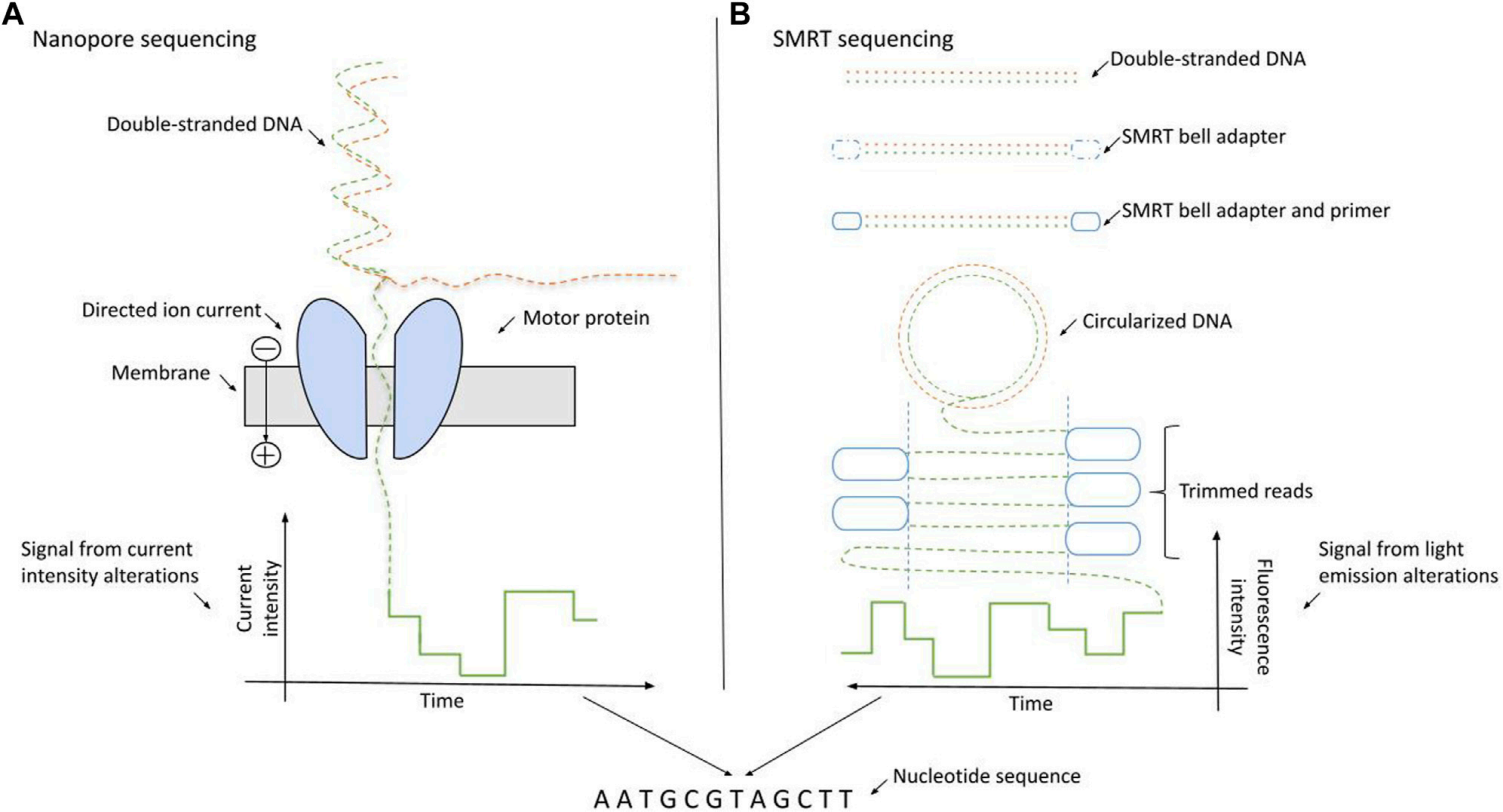
Schematics of long-read sequencing approaches: nanopore sequencing (Oxford Nanopore Technologies) and SMRT sequencing (Pacific BioSciences). **(A)** NS relies on the passage of the ssDNA through the membrane driven by the motor protein. The nucleotide bases are identified using the directed ionic current intensities arising from the motion of particles through the membrane. **(B)** During SMRT sequencing, dsDNAs are circularized into a SMRT bell. The SMRT bell contains the four fluorescently labeled nucleotides with unique emission spectra. The bases are distinguished by the alterations in the light emission spectra.

The nanopore sequencing (NS) method, distributed by Oxford Nanopore Technologies (ONT), is based on the detection of the electric current changes provoked by the disorganization of nanopore proteins [16–19]. The alterations in the real-time produced electric current can be measured directly. During NS, dsDNA molecules are denatured, and the motor protein directs ssDNAs through the nanochannels (pores) one after the other. The passage of ssDNA molecules leads to disturbances in the electric current, which is detected by specific reader sensor proteins. The deflections are distinct for all nucleotides resulting in unique signatures for each base. The entire process happens inside a device-specific flow cell [20], which contains thousands of nanopore channels. The schematics of NS sequencing is presented on Figure 1A.

Since the release of the first ONT sequencing device—named MinION—in the mid-2010s, the continuous improvement of the key factors, like accuracy, read length, and sequencing throughput is present. The throughput is determined by the number of active pores on the flow cells and by the DNA/RNA translocation speed. To provide the maximal amount of available active pores on the flow cells, their periodical revision is secured [16, 21]. The read length and the accuracy are highly dependent on the released version and quality of the sequencing chemistry—which in this case includes the traits of nanopores and motor proteins,—however, by introducing special adapters during penetration, an increase in accuracy measure can also be reached with higher ∼420 bps per sec sequencing speeds compared to the previous ∼70 bps per sec rate [22].

NS reads are characterized by longer lengths of 10 kb up to 100 kb, which means more sequenced bases, more generated data and increased informatics resource needs compared to NGS. The large amount of data means longer bioinformatics analysis time and more expensive informatics hardware park. However, due to the increased information amount, a more accurate identification of alterations becomes available. As the most important disadvantage of the increased generated data, higher error rate and read misclassification can be experienced on the ONT platforms compared to NGS [23, 24].

### SMRT sequencing

Pacific BioSciences provided the first nanosensor-based technology in the early 2010s relying on the single molecule real-time (SMRT) sequencing model [25]. The key factor in this method is the detection of alterations in light emission when the DNA polymerase incorporates a nucleotide [26]. In more detail, SMRT sequencing is done by the immobilization of the DNA polymerase in each well of a special silicone chip (SMRTcell) using DNA as the mobile molecule. DNA templates are presented as closed, single-stranded DNA (ssDNA) molecules, named SMRTbells, which are created by ligating hairpin adaptors to both ends of a target double-stranded DNA (dsDNA). The SMRTcells contain four fluorescently labeled nucleotides with unique emission spectra. Zero mode waveguides (ZMW) are optical waveguides developed for rapid light sensing and provide the interface for the detection of light emitted by the incorporation of phosphate-labeled dNTPs of SMRTcells [27, 28]. The process of SMRT sequencing is illustrated on Figure 1B.

Compared to NGS, the precision of SMRT sequencing is lower, as an example, due to the many inaccuracies during base identification. Although, in contrast to the experienced higher error rates and costs-per-base, the technology grants several orders of magnitude increase in read lengths (few Mbps in contrast to the previous few hundred bps) and faster sequencing runs. As a possibility, the arising conflict regarding the advantages and disadvantages of NGS and SMRT sequencing suggests the consideration of hybrid-sequencing solutions in the future. Hybrid units are the combinations of different sequencing methods and can be promising to overcome the deficiencies [29].

## Technical advances and difficulties of long reads

Following a brief histological and methodological overview of long-read approaches, we detail the technical background and give comprehensive knowledge regarding sequencing challenges and advances. Compared to NGS approaches, the main difficulties of TGS are the overall lower per read accuracy and poorer read quality [30]. In contrast to short reads, long reads are much noisier. Prolonged lengths induce an increase in the number of bases and in reading time. Both contribute to a higher probability of collecting false information, promoting more noise and uncertainty [31]. The continuous change of the sequencing reads in length during a single run also indicates the higher chance of inaccuracies. Due to the above-listed reasons, the proper handling of deflections cannot be emphasized enough and the problem-concentrated improvements are published continuously [32]. Although in the early times base identifying accuracy was around 85% (indicating the error rate to be nearly 15%), nowadays almost 99% (SMRT) and 95% (NS) can be reached [31, 33]. Error correction methods [34, 35] provide a solution to resolve the inaccuracies and are divided into two groups: hybrid and non-hybrid approaches [36]. Hybrid methods take the advantage of the high accuracy of short reads for correcting errors in the long threads, while non-hybrid methods perform self-correction with long reads using overlap information. The effectiveness of error correction methods is highly dependent on the sequencing coverage [36], thus shows a dependence on the percentage of all sequenced base pairs or loci of the genome.

In SMRT devices, the read quality is proportional to the number of DNA fragment transitions. For example, the reading accuracy is around 85%–87% in a 10 kb long sequence if it is passing only once [37]; however, with multiple reading, it can be further improved reaching 99%. In contrast, the quality of NS reads is independent of the reading repetition times and the length of nucleic acid sequences. It only depends on the ratchet rate per base through the nanopores. Fragments traverse only once, the median sign-pass accuracy is around 95% [38], and read length depends only on the amount and the quality of the high-molecular weight input DNA. To reach the maximal sequencing precision, companies focusing on long-reads tend to release chemistry, software, and hardware updates regularly [16, 39].

Reference genomes are integral parts of sequencing assays as they provide the organism-specific support during base order construction [40]. The progression of sequencing methods derived the breakthroughs regarding the imprecision of reference genomes [41], variant identification, genomic assemblies, and other specialized data analyses in the field of genetics. The Genome Reference Consortium (GRC) released the current form of human reference genome (GRCh38. p13) in 2013 with an origin tracing to the Human Genome Project [42, 43]. In contrast to the continuous improvement of the GRCh38.p13 genome, over the last years, due to the technical limitations of NGS short reads, many problems remained unsolved. The underrepresentation of repetitive sequences, the unsolved assembly gaps due to structural polymorphisms and the unfinished polymorphic regions resulted in the need of further investigation. The 151 mega-base (Mbp) pair long unknown sequence data distributed throughout the GRCh38. p13 genome turned out to be fundamental and included centromere and telomere regions, segmental duplications, amplicon gene arrays and ribosomal DNA (rDNA) arrays, all highly affecting cellular processes [44]. Long-read sequencing proved to be the problem-solver, indicating the birth of the Telomere-to-Telomere (T2T) Consortium to construct a new and almost complete human reference genome, the T2T-CHM13 assembly [44]. In this cooperation, the advances of long-read techniques, including the multi-kilobase single-molecule reads of SMRT and the ultra-long reads of NS were combined, providing evidence to the beneficial applications of hybrid sequencing methods. The T2T-CHM13 assembly resulted in a 3 billion-base pair long complete human haplotype, contributing to the recognition of almost 4,000 new genes, with high rates of protein coding nature. In addition, T2T-CHM13 includes the gapless telomere-to-telomere assemblies for all 22 human autosomes and chromosome X, contains the corrected version of the 151 Mbp long unknown genomic sequence data, and has the chance to arise as the mainly applied reference genome in human genomics-related fields. The successful application of the combination of NS and SMRT reads as a hybrid solution in the T2T Consortium projects that the further development of sequencing methods can be still expected, and the seeking to eliminate their limitations is continuous.

## Bioinformatics of long reads

After exploring the scientific literature in detail, it clarified that sequencing techniques cannot address questions in genomics without bioinformatics. With the rise of new sequencing approaches, a new generation of bioinformatics tools emerged, being compatible with the unique features of long reads and trying to overcome their biases. As long reads, their analysis also presented many opportunities and challenges. Increased read lengths particularly affects how aligners, assemblers, variant callers store and analyze the data. Many software tools specialized for long-read sequencing data are provided by ONT and PacBio with continuous monitorization [45, 46]. Additional sources and packages are also presented, as it is demonstrated in Table 1.

**TABLE 1:** Summary of the most recent and common-used long-read bioinformatics tools.

Long-read bioinformatics tools				
	Data analysis step	Tool name	Background and performance	References
Complex user-friendly interfaces capable of perform the whole analysis process exept error correction: PacBio: SMRT link (BioSciences) Nanopore: EPI2ME Labs (Nanopore)	QC metrics	FastQC, MultiQC, LongQC, NanoPack, MinIONQC, NanoR, RNASeQC	The listed items are quality control (QC) tools suitable for sequencing approaches, including long- and short-reads. Their aim is to provide QC checks on raw sequence data (FastQC) or dataset (MultiQC) and give detailed feedback regarding the occurring problems. For RNA-seq data, an unique algorithm (RNA-SeQC) was developed	[47–54]
	Base calling	SMRT analysis tools, Dorado, Guppy	Neural network and statistical method based base calling methods; SMRT reads require specific analysis tools. Dorado and Guppy were developed for NS reads	[55–57]
	Variant calling	Clair3, Sniffles	Sniffles perform structural variant calling on noisy long-read data. Clair3 is a deep neural network based variant caller even capable of haplotype-sensitive variant detecion performing variant detection from sequencing data containing modified bases	[58–60]
		wf-human-variation, wf-somatic-variation	Complex command line compatible workflows for NS variant detection. On demand, the separate or combined usage of tumor and normal data is insured with the production of well-detailed analysis reports	[61]
	Modified base calling	Modbamtools, Guppy, Mekada, DeepSignal, DeepMod	Set of tools to manipulate and visualize DNA/RNA base modification and methylation data that are stored in.bam format. Some of them is suitable for all long-read techniques. The detectable modified bases are 5mC, 5hmC and 6 mA	[33, 57–59, 62, 63]
	Genome assembly	Flye, Canu, HiCanu, BLASR, FALCON	Some of them are graph construction-based method (Flye) or using hierarchical genome assembly process with clustering (BLASR) and overlap-based error correction, also carry out phasing (FALCON) during the accomplishment of *de novo* genome assembly on high-noise single-molecule sequencing data	[64–68]
	Visualization	NanoPack, R packages: maftools, ggplot2, Python packages: matplotlib (pyVolcano)	Packages offering universal and problem-specific solutions for long-read data visualization	[50, 69–72]
	Error correction	Pilon, Racon, DeepConsensus, Medaka	Neural network- and transformer-based methods, which are intended as standalone modules to correct raw contigs generated by rapid assembly methods which include or do not include a consensus step. An advantage of the application of transformer-based error correction methods is that they leverage a unique alignment loss to correct sequencing errors	[33, 35, 71]

Additional packages are listed on webpage https://long-read-tools.org and can be found on bioinformatics-related pages.

As a summary of bioinformatics steps, the following section will provide a brief general discussion regarding base calling, detection of base modifications, variant calling, genome assembly, and a bit of specialized evaluation possibilities including both long-read and NGS techniques, emphasizing their unique prominences.

### Base calling

The first main step in bioinformatics analysis is always a process named base calling during which the specific electric signals are translated into known nucleotides. The phrase of translation in this case means the conversion process from electric signals to nucleic acid sequences [73]. Raw current and light pulse data and read information are stored in specific format files. In the NGS system, the primary analysis of sequencing data is a critical step before base calling. These sequencing platforms have their own chemical- or sensor-origin biases which should be eliminated before or during base calling [74]. As a result of the pre-sequencing PCR amplification, many redundant PCR duplicates are present among aligned reads, which are marked and excluded in later analysis stages [75]. Considering the two long-read techniques, base calling means the conversion of fluorescent light pulses in SMRT devices, while during NS, the translation of current intensities into k-mers of bases. The alignment of sequencing reads to a reference sequence is a compulsory step after base calling in NGS bioinformatics, however many TGS base callers [55–57] execute the alignment in parallel with base identification [55, 56]. As a side note, we would like to emphasize the importance of quality check of sequencing reads [47–54, 76, 77] preferably before and after every principal step, paying special attention to base calling and variant calling.

### Epigenetic modifications: modified base calling

In addition to traditional bases, like adenine (A), thymine (T), uracil (U), guanine (G) and cytosine (C), DNA and RNA molecules can contain modified bases that alter from their original mates in nature and frequency and have different functional roles. In nucleic acids, the most frequently occurring modified bases are 6mA, 5mC, and 5hmC. Considering the location of 5mC and 5hmC in DNA, they are mostly observed on CpG dinucleotide sites. RNA modifications, including 6mA, are frequent in non-coding RNA like ribosomal RNA (rRNA), transfer RNA (tRNA), and also in coding mRNA. Modified DNA and RNA nucleotides play a key role in many biological processes including development, aging, and cancer [78–80]. Their identification secures the analysis of open chromatin regions, the detection of DNA replication and the measurement of RNA metabolism using base analogs [81–83].

The methylation signature is not preserved in PCR amplification—which is essential before NGS assays -, thus approaches have been developed to conserve the epigenetic information. These pretreatments rely on methylation-dependent enzymatic restriction, methyl-DNA enrichment, and direct bisulfite conversion [84]. In NGS base modification analysis bisulfite-treated DNAs require specialized alignment to account for the C to T conversion. Encouraged by this, short read alignment algorithms were implemented that can be configured for bisulfite-converted DNA alignment [85].

However, the available NGS methods provided some sort of identification of modified bases in nucleic acid sequences as well, but the real landscape demonstration became fulfilled with TGS assays. The detection of modified bases in SMRT is based on the delay between fluorescence pulses [86]. NS relies on the recognition of the signal shifts resulting from the different current flow through nanopores [19, 87]. Most TGS computational tools are capable of modified base detection from reference-aligned reads [34, 57–59, 62, 63, 88], and are based on machine learning training models and statistical tests. Algorithms using neural networks show the highest performance, although statistics-based approaches are the best suited for the identification of *de novo* modifications [34, 89]. Because of software developmental progress, long-read base callers became capable of calling modified bases directly [55, 56]. The key is the application of specific base calling configuration models indicating in their labels the name of the modified bases of interest [56].

### Variant calling

Sequence variations can be grouped based on their somatic or germline nature. Germline variants are presented in all cells of the body, including the germ cells, while somatic mutations arise during lifetime. The standard pipeline of somatic mutation calling is the paired tumor-normal sequencing strategy [90]. It can provide the true somatic mutations by filtering out the germlines of the normal from the tumor mutation data according to some known tissue-specific non-tumorous variant profiles. Germline and somatic groups also involve subtypes like structural variants, single nucleotide variations, short insertions/deletions, and copy number variations.

The shortest variations are single nucleotide polymorphisms, which are germline substitutions of single nucleotides at specific genomic positions. Copy number variation (CNV) is an alteration type describing the uniqueness among individual genomes, meaning a few and thousands of base-scale variations in the copy numbers of specific DNA segments. SVs are large genomic alterations, like insertions, deletions, inversions, and translocations. They are typically longer than 50 bp, describing different combinations of DNA losses, gains, or rearrangements [91]. Structures shorter than 50 bp and longer than few bases are usually referred to as indels.

The key aspect of variant calling is the choice of a robust variant caller concerning NGS and TGS assays as well. To achieve the optimal performance, a prior fine-tuning considering the features of the input is needed. This optimal performance is reached by training and pre-testing the variant callers using the characteristics of the datasets. The exclusion of redundant and duplicate reads from binary alignment mapping (.bam) files, the quality control of .bams, and the identification and the reduction of false-positive variant calls caused by alignment artifacts are crucial steps in input preparation. The accuracy of variant calling can be validated by benchmarking datasets, which are publicly available. The quality of the collected variants is dependent on the precision (and version) of the reference genome, and on the error rate and accuracy of the base and variant identification method. Sequencing coverage affects the sensitivity in a hidden manner, since the appropriateness of the variant caller input is highly dependent on the coverage [92]. We must consider the variant representation differences when searching valid variations from the reference by excluding the low coverage b(i)ases. The appropriate post-filtering of the output data is often required; it prevents us from artificial and false-positive calls [75].

TGS variant callers [58–61, 88] are built upon *de novo* assembly, short-read alignment, or long-read mapping approaches. *De novo*-assembled sequences cover the alignment of the current assembly to another, or to a reference sequence, and the alterations can be identified by a pointwise positional comparison. During short-read alignment, the presence of SVs induces the appearance of abnormally oriented and spaced reads replacing the organized paired-end form. Long-read mapping approaches can span repetitive and other problematic regions simply, showing an overall better performance [93].

In nowadays-used techniques, long-read sequencing is the most suitable and the most accurate variant calling approach, but especially for the detection of structural variants (SVs) [94]. The special role of genetic variations, especially SVs, has been highlighted primarily in medicine and molecular biology, e.g., in neurological diseases [95, 96], or during the detection of oncogene-specific variations in breast, prostate, or primary gastric tumors [97]. Although their importance is unquestionable, they have been understudied in the past. The origin of this issue arises from the fact that they can overlap or be nested giving rise to complex patterns, which are hard to identify with short-read approaches [93].

### Genome assembly

Probably the most important benefit of long-read computational biology can be experienced in the fields of genomic *de novo* assemblies [64–68]. The phrase assembly in this case means the comparison and coupling of the read sequences to each other. Assembly construction is crucial to understand the impact of genomic diversity on health and disease [98]. In the last few years, the process has been simplified and the results are more accurate due to the improvements in the bioinformatics routines [99]. Besides the sequence construction, another important application of genomic assemblies is the reassembling and fixing of the errors of former reference genomes (of fungal, plant, animal, and human) [44]. Unfortunately, repetitive sequences with unresolved repeats are still problematic, enhancing confusion while joining assembled sequences. Linked sequences contain many gaps. To get rid of these, the scaffolding of sequences is a crucial aspect. The term scaffolding means the proper ordering and orientation of assembled sequences using genetic markers, optical maps, or linked reads [100]. Assemblies of the short and long reads are both presented taking their advantages in different issues. Besides the success of their combination in T2T Consortium, many other hybrid applications have been published recently [29, 101], invoking that for accurate genomic assemblies we need error-free short and long sequences.

## Applications of long-read sequencing

Although the topic of long-read sequencing is quite recent, its successful application in several fields is highly presented in the scientific literature including cancer genomics, laboratory medicine, methylation studies and rare genetic conditions, as well.

In laboratory medicine, the currently applied diagnostic strategies involve the use of targeted NGS gene panels, exome sequencing, and genome sequencing. Targeted gene panels are somatic and hereditary disease-specific with the ability to maximize coverage, sensitivity, and specificity of characteristic genes. They offer higher diagnostic yield thanks to lower costs and faster diagnostic times, than exome or genome sequencing [102]. The combination of whole-genome and long-read targeted sequencing has already been applied in hematology. Hematologic disorders, like hemophilia A, often involve the appearance of gene fusions and other pathologic events, thus the characterization of fusion transcripts is often done by the combination of NGS and TGS assay-based methods [103]. Another example of laboratory medicine related application of long reads is the characterization of the human leukocyte antigen (HLA) system. The HLA system contains the genes that encode key components of the adaptive immune system, and accounts for the major genetic differences among ethnic populations [104]. HLA-genotyping information is often yielded from targeted exome and non-targeted genome sequence data [105].

For diploid genomes, chromosomal DNA has two haplotypes. These are combinations of alleles from multiple genetic loci on the same chromosome including complex structural variants, one inherited from each parent. Distinguishing the maternal and paternal haplotypes allows the recognition of homozygous and heterozygous mutations in the human genome. Haplotypes within a diploid chromosome are determined by finding a partitioning of reads to two sets, one for each haplotype, such that the reads within subsets have a minimal number of errors compared to a consensus [106]. Their presence helps to discover the nested structural variations, inversions, and other complex rearrangements and studies the interactions between variants in regulatory elements, aneuploidy, evolutionary processes, and drug resistance in viral infections. The key concept to derive haplotypes using sequencing reads is the phasing of heterozygous variants. The advancements in sequencing associated computational tools like reference-based phasing, *de novo* assembly, or strain-resolved metagenome assembly [107] entail the potential for the near-complete human haplotype structure reconstruction.

The investigation of genomes containing segments with small allele fraction variants and observed rearrangements in regions of associated genes is still challenging even for current long-read methods [108]. The appearance of sequencing techniques with higher-depths and longer-lengths is expected. Regardless, many successful applications can be discussed already. The characterization of tumor genomes and transcriptomes with the analysis of mRNA expression, mutation detection, gene fusions, or chromosomal copy number alterations can highlight new markers of malignancy. With better depiction of the genome-wide landscape and the extent of mutational processes, whole-genome long-read sequencing yields better treatment options in advanced thyroid [109] and other cancers [110]. Improvements in sequencing technologies allowed the recognition and the description of long non-coding RNAs (lncRNAs). They are non-protein coding nucleic acids with lengths greater than 200 nucleotides and characterized by high cell type specificity [111]. LncRNAs are found to be key players in tumorigenesis and immune responses, and evidence supports their unique cellular functions in the tumor immune microenvironment [112]. Most studies related to lncRNAs relied on bulk RNA-sequencing; however, the potentials of scRNAseq can open new possibilities to understand the cell type-specific functions of lncRNA genes [112].

The examination of abnormal RNA expressions helps to understand the molecular mechanisms behind human cancer initiation, development, progression, and metastasis. RNA techniques include the classic bulk RNA (RNAseq), the single-cell RNA (scRNAseq), the spatial RNA (spRNAseq) [113] and the direct RNA (DRS) [114] sequencing methods. Bulk RNAseq means the sequencing of mRNA-only or whole transcriptome libraries with single-end short or paired-end longer approaches. scRNAseq procedures always include single-cell isolation and capture, cell lysis, reverse transcription, cDNA amplification, and library preparation [115]. spRNAseq combines the transcriptional analysis of bulk RNAseq and *in situ* hybridization providing whole transcriptome data with spatial information [113]. As a novelty, NS terminology offers the direct sequencing of individual polyadenylated RNAs without the need of any amplification step [114].

Circulating cell-free DNA (cfDNA) in the blood of cancer patients can be the signal of worsening tumor progression. Sequencing analyses revealed that tumor-derived cfDNA accounts for only a fraction of the total amount of cfDNAs and this fraction varies according to the tumor burden [116]. Due to the low level and high fragmentation of cfDNAs, their analysis is challenging. In the past few years, NGS techniques were suitable tools for this assay [117], however, the long-reads will possibly promote the provision of deeper cfDNA characteristics providing higher clinical sensitivity for the detection of cancers [117].

The clinical diagnosis of rare genetic disorders often requires the identification of CNVs or repeat variants. Long-read genome sequencing provides an improved opportunity for CNV detection and broadens the possibilities of gene and variant level annotation [118]. As an interesting example, primary mitochondrial diseases (PMD) comprise a group of rare genetic conditions characterized by impaired mitochondrial oxidative phosphorylation. The presence of mixed populations of mitochondria, named heteroplasmy, and the fact that those mitochondria contain its own genome consisting of mitochondrial DNA (mtDNA) poses a challenge in identifying PMD. Long-read sequencing enables the entire mitochondrial genome to be sequenced in one read, ensuring the overcome of the obstacles mentioned-above [119].

Using epigenetic alterations as biomarkers presents a unique opportunity for early cancer detection, monitoring, and prognosis. Methylation is the most widely studied epigenetic modification of nucleic acids and its landscape in cancer tissues is evidently complex and highly variable. DNA methylation plays an important role in the regulation of gene expression. The methylation-associated transcriptional inactivation of genes involved in cell cycle control and damage repair suggest that aberrant nucleotide methylations are hallmarks of carcinogenesis [120, 121]. NS provides the most precise detection and description of methylation landscapes [122]. Studies showed that the methylation of both 5mC and 5hmC has a role in the pathogenesis of pediatric cancer [123], while the presence of 6mA in pancreatic tumors is highly upregulated and has a lower occurrence compared to 5mC [124]. Thus, the idea to use methylation as a biomarker for cancer detection is not far to seek. Due to its prognostic property, DNA methylation was already applied as a prognostic marker in several cancer types, including prostate, bladder, colorectal, non-small-cell lung, breast, ovarian, cervical cancer, and liver malignancies [125, 126].

Although we presented the potentials of TGS long-read sequencing, their utilization in routine diagnostics has not widespread yet. NGS whole exome and targeted sequencing techniques offer well applicable results in routine diagnostics including inborn discrepancy detection, cancer research and diagnostics, hematology, and neurological disorders [72, 127–130]. Their instrumentation, the corresponding chemicals, and flow cells are more affordable, and the generated data are more targeted [131]. On the other hand, as long-read techniques offer a wider genomic picture, thus providing a deeper insight into nucleic acid traits, their introduction into routine examinations has started [132–136] and their spreading is expected in the near future.

## Conclusion

In this review, we discussed the milestones of sequencing techniques, their progression, current applications, and future opportunities. We also provided a general comparison between short- and long-read assays highlighting their strengths and drawbacks from various aspects including methodology, data analysis, and applications. As we introduced in the last chapter, the spread of long-read techniques has led to a rapid progress in genomics-related areas. By expanding and refining sequencing routines, it becomes possible to explore the genetic complexity of biological systems in greater depths facilitating a radical future advance in the field of sequence variances.

## References

[1] Alberts B. 4th chapter: DNA, chromosomes and genomes. In: Molecular biology of the cell. 6th ed. W.W. Norton & Company (2015).

[2] Adewale BA. Will long-read sequencing technologies replace short-read sequencing technologies in the next 10 years? Afr J Lab Med (2020) 9(1):1340. 10.4102/ajlm.v9i1.1340 33354530 PMC7736650

[3] Sanger F Nicklen S Coulson AR. DNA sequencing with chain-terminating inhibitors. Proc Natl Acad Sci USA (1977) 74(12):5463–7. 10.1073/pnas.74.12.5463 271968 PMC431765

[4] Maxam AM Gilbert W. A new method for sequencing DNA. Proc Natl Acad Sci USA (1977) 74(2):560–4. 10.1073/pnas.74.2.560 265521 PMC392330

[5] Marulies M Egholm M Altman WE Attiya S Bader JS Bemben LA Genome sequencing in microfabricated high-density picolitre reactors. Nature (2005) 437:376–80. 10.1038/nature03959 16056220 PMC1464427

[6] Guo J Yu L Turro NJ Ju J. An integrated system for DNA sequencing by synthesis using novel nucleotide analogues. Acc Chem Res (2010) 43(4):551–63. 10.1021/ar900255c 20121268 PMC2857541

[7] Liu L Li Y Li S Hu N He Y Pong R Comparison of next-generation sequencing systems. J Biomed Biotechnol (2012) 2012:251364. 10.1155/2012/251364 22829749 PMC3398667

[8] Harrington CT Lin EI Olson MT Eshleman JR. Fundamentals of pyrosequencing. Arch Pathol Lab Med (2013) 137(9):1296–303. 10.5858/arpa.2012-0463-RA 23991743

[9] Grigorev K Foox J Bezdan D Butler D Luxton JJ Reed J Haplotype diversity and sequence heterogeneity of human telomeres. Genome Res (2021) 31(7):1269–79. 10.1101/gr.274639.120 34162698 PMC8256856

[10] Kumar KR Cowley MJ Davis RL. Next-generation sequencing and emerging technologies. Semin Thromb Hemost (2019) 45(7):661–73. 10.1055/s-0039-1688446 31096307

[11] Chen X Xu H Shu X Song CX. Mapping epigenetic modifications by sequencing technologies. Cell Death Differ (2023). 10.1038/s41418-023-01213-1 PMC11742697 37658169

[12] Xiao T Zhou W. The third generation sequencing: the advanced approach to genetic diseases. Transl Pediatr (2020) 9(2):163–73. 10.21037/tp.2020.03.06 32477917 PMC7237973

[13] Athanasopoulou K Boti MA Adamopoulos PG Skourou PC Scorilas A. Third-generation sequencing: the spearhead towards the radical transformation of modern genomics. Life (Basel) (2021) 12(1):30. 10.3390/life12010030 35054423 PMC8780579

[14] Kaplun L Krautz-Peterson G Neerman N Stanley C Hussey S Folwick M ONT long-read WGS for variant discovery and orthogonal confirmation of short read WGS derived genetic variants in clinical genetic testing. Front Genet (2023) 14:1145285. 10.3389/fgene.2023.1145285 37152986 PMC10160624

[15] Roberts RJ Carneiro MO Schatz MC. The advantages of SMRT sequencing. Genome Biol (2013) 14(7):405. 10.1186/gb-2013-14-6-405 23822731 PMC3953343

[16] ONT Nanopore Technologies. Continuous development and improvement (2023). Available from: https://nanoporetech.com/about-us/continuous-development-and-improvement (Accessed 2024).

[17] Pollard MO Gurdasani D Mentzer AJ Porter T Sandhu MS. Long reads: their purpose and place. Hum Mol Genet (2018) 27(R2):R234–R241. 10.1093/hmg/ddy177 29767702 PMC6061690

[18] Quick J Loman NJ. Nanopore sequencing: an introduction. World Scientific Press (2019).

[19] Deamer D Akeson M Branton D. Three decades of nanopore sequencing. Nat Biotechnol (2016) 34:518–24. 10.1038/nbt.3423 27153285 PMC6733523

[20] ONT Nanopore Technologies. Flow cells (2023). Available from: https://nanoporetech.com/how-it-works/flow-cells-and-nanopores (Accessed 2023).

[21] Nicholls SM Quick JC Tang S Loman NJ. Ultra-deep, long-read nanopore sequencing of mock microbial community standards. GigaScience (2019) 8(5):giz043. 10.1093/gigascience/giz043 31089679 PMC6520541

[22] Ni Y Liu X Simeneh ZM Yang M Li R. Benchmarking of Nanopore R10.4 and R9.4.1 flow cells in single-cell whole-genome amplification and whole-genome shotgun sequencing. Comput Struct Biotechnol J (2023) 21:2352–64. 10.1016/j.csbj.2023.03.038 37025654 PMC10070092

[23] Jennings W. Illumina sequencing (2016). 10.1201/9781315181431-7

[24] StefanCPHallATGrahamASMinogueTD. Comparison of illumina and Oxford nanopore sequencing technologies for pathogen detection from clinical matrices using molecular inversion probes. J Mol Diagn (2022) 24(4):395–405. 10.1016/j.jmoldx.2021.12.005 35085783

[25] Harris TD Buzby PR Babcock H Beer E Bowers J Braslavsky I Single-molecule DNA sequencing of a viral genome. Science (2008) 320(5872):106–9. 10.1126/science.1150427 18388294

[26] Eid J Fehr A Gray J Luong K Lyle J Otto G Real-time DNA sequencing from single polymerase molecules. Science (2009) 323(5910):133–8. 10.1126/science.1162986 19023044

[27] Levene MJ Korlach J Turner SW Foquet M Craighead HG Webb WW. Zero-mode waveguides for single-molecule analysis at high concentrations. Science (2003) 299(5607):682–6. 10.1126/science.1079700 12560545

[28] Garrido-Cardenas JA Garcia-Maroto F Alvarez-Bermejo JA Manzano-Agugliaro F. DNA sequencing sensors: an overview. Sensors (Basel) (2017) 17(3):588. 10.3390/s17030588 28335417 PMC5375874

[29] Vasudevan K Devanga Ragupathi NK Jacob JJ Veeraraghavan B. Highly accurate-single chromosomal complete genomes using IonTorrent and MinION sequencing of clinical pathogens. Genomics (2020) 112(1):545–51. 10.1016/j.ygeno.2019.04.006 30978388

[30] Warburton PE Sebra RP. Long-read DNA sequencing: recent advances and remaining challenges. Annu Rev Genomics Hum Genet (2023) 24:109–32. 10.1146/annurev-genom-101722-103045 37075062

[31] Ebler J Haukness M Pesout T Marschall T Paten B. Haplotype-aware diplotyping from noisy long reads. Genome Biol (2019) 20:116. 10.1186/s13059-019-1709-0 31159868 PMC6547545

[32] Delahaye C Nicolas J. Sequencing DNA with nanopores: troubles and biases. PLoS One (2021) 16(10):e0257521. 10.1371/journal.pone.0257521 34597327 PMC8486125

[33] Amarasinghe SL Su S Dong X Zappia L Ritchie ME Gouil Q. Opportunities and challenges in long-read sequencing data analysis. Genome Biol (2020) 21:30. 10.1186/s13059-020-1935-5 32033565 PMC7006217

[34] Liu Q Fang L Yu G Wang D Xiao CL Wang K. Detection of DNA base modifications by deep recurrent neural network on Oxford Nanopore sequencing data. Nat Commun (2019) 10(1):2449. 10.1038/s41467-019-10168-2 31164644 PMC6547721

[35] Baid G Cook DE Shafin K Yun T Llinares-López F Berthet Q DeepConsensus: gap-aware sequence transformers for sequence correction. Nat Biotechnol (2023) 41(2):232–8. 10.1038/s41587-022-01435-7 36050551

[36] Zhang H Jain C Aluru S. A comprehensive evaluation of long read error correction methods. BMC Genomics (2020) 21(6):889. 10.1186/s12864-020-07227-0 33349243 PMC7751105

[37] Ardui S Ameur A Vermeesch JR Hestand MS. Single molecule real-time (SMRT) sequencing comes of age: applications and utilities for medical diagnostics. Nucleic Acids Res (2018) 46(5):2159–68. 10.1093/nar/gky066 29401301 PMC5861413

[38] ONT Nanopore Technologies. Clive Brown’s keynote at nanopore community meeting (2018). Available from: https://nanoporetech.com/resource-centre/clive-brown-ncm-2018 (Accessed 2018).

[39] Pacific BioSciences HiFi sequencing (2023). Available from: https://www.pacb.com/technology/hifi-sequencing/ (Accessed 2024).

[40] Completing Human Genomes. Completing human genomes. Nat Methods (2022) 19:629. 10.1038/s41592-022-01537-9 35689025

[41] Goodwin S Gurtowski J Ethe-Sayers S Deshpande P Schatz MC McCombie WR. Oxford Nanopore sequencing, hybrid error correction, and *de novo* assembly of a eukaryotic genome. Genome Res (2015) 25(11):1750–6. 10.1101/gr.191395.115 26447147 PMC4617970

[42] Hood L Rowen L. The Human Genome Project: big science transforms biology and medicine. Genome Med (2013) 5:79. 10.1186/gm483 24040834 PMC4066586

[43] Lander ES Linton LM Birren B Nusbaum C Zody MC Baldwin J Initial sequencing and analysis of the human genome. Nature (2001) 409:860–921. 10.1038/35057062 11237011

[44] Nurk S Koren S Rhie A Rautiainen M Bzikadze AV Mikheenko A The complete sequence of a human genome. Science (2022) 376:44–53. 10.1126/science.abj6987 35357919 PMC9186530

[45] Suzuki Y. Informatics for PacBio long-reads. Single molecule and single cell sequencing. In: Suzuki Y, editor. Advances in experimental medicine and biology. Springer (2019). 10.1007/978-981-13-6037-4_8 30968364

[46] Oxford Nanopore Technologies. Oxford nanopore community (2023). Available from: https://nanoporetech.com/community (Accessed 2024).

[47] Bioinformatics. Babraham bioinformatics (2023). Available from: https://www.bioinformatics.babraham.ac.uk/projects/fastqc/ (Accessed 2023).

[48] Ewels P Magnusson M Lundin S Käller M. MultiQC: summarize analysis results for multiple tools and samples in a single report. Bioinformatics (2016) 32(19):3047–8. 10.1093/bioinformatics/btw354 27312411 PMC5039924

[49] Fukasawa Y Ermini L Wang H Carty K Cheung MS. LongQC: a quality control tool for third generation sequencing long read data. G3 Genes, Genomes, Genet (2020) 10(4):1193–6. 10.1534/g3.119.400864 PMC7144081 32041730

[50] Coster WD D'Hert S Schultz DT Cruts M Van Broeckhoven C. NanoPack: visualizing and processing long-read sequencing data. Bioinformatics (2018) 34(15):2666–9. 10.1093/bioinformatics/bty149 29547981 PMC6061794

[51] Lanfear R Schalamun M Kainer D Wang W Schwessinger B. MinIONQC: fast and simple quality control for MinION sequencing data. Bioinformatics (2019) 35(3):523–5. 10.1093/bioinformatics/bty654 30052755 PMC6361240

[52] Bolognini D Bartalucci N Mingrino A Vannucchi AM Magi A. NanoR: a user-friendly R package to analyze and compare nanopore sequencing data. PLoS One (2019) 14(5):e0216471. 10.1371/journal.pone.0216471 31071140 PMC6508625

[53] Graubert A Aguet F Ravi A Ardlie KG Getz G. RNA-SeQC 2: efficient RNA-seq quality control and quantification for large cohorts. Bioinformatics (2021) 37(18):3048–50. 10.1093/bioinformatics/btab135 33677499 PMC8479667

[54] PacBio SMRT^®^. Tools reference guide (v11.0) (2022). Available from: https://www.pacb.com/wp-content/uploads/SMRT_Tools_Reference_Guide_v11.0.pdf (Accessed 2022).

[55] Oxford Nanopore Technologies. Oxford nanopore technologies (2023). Available from: https://github.com/nanoporetech/dorado (Accessed 2023).

[56] Oxford Nanopore Technologies. Oxford nanopore technologies (2024). Available from: https://community.nanoporetech.com/docs/prepare/library_prep_protocols/Guppy-protocol/v/gpb_2003_v1_revax_14dec2018/guppy-software-overview (Accessed 2024).

[57] Zheng Z Li S Su J Leung AWS Lam TW Luo R. Symphonizing pileup and full-alignment for deep learning-based long-read variant calling. Nat Comput Sci (2022) 2(12):797–803. 10.1038/s43588-022-00387-x 38177392

[58] Sedlazeck FJ Rescheneder P Smolka M Fang H Nattestad M von Haeseler A Accurate detection of complex structural variations using single-molecule sequencing. Nat Methods (2018) 15:461–8. 10.1038/s41592-018-0001-7 29713083 PMC5990442

[59] Romagnoli S Bartalucci N Vannucchi AM. Resolving complex structural variants via nanopore sequencing. Front Genet (2023) 14:1213917. 10.3389/fgene.2023.1213917 37674481 PMC10479017

[60] Oxford Nanopore Technologies. Oxford nanopore technologies (2018). Available from: https://github.com/nanoporetech/medaka (Accessed 2018).

[61] Ni P Huang N Zhang Z Wang DP Liang F Miao Y DeepSignal: detecting DNA methylation state from Nanopore sequencing reads using deep-learning. Bioinformatics (2019) 35(22):4586–95. 10.1093/bioinformatics/btz276 30994904

[62] Kolmogorov M Yuan J Lin Y Pevzner P. Assembly of long error-prone reads using repeat graphs. Nat Biotechnol (2019) 37(5):540–6. 10.1038/s41587-019-0072-8 30936562

[63] Koren S Walenz BP Berlin K Miller JR Bergman NH Phillippy AM. Canu: scalable and accurate long-read assembly via adaptive k-mer weighting and repeat separation. Genome Res (2017) 27(5):722–36. 10.1101/gr.215087.116 28298431 PMC5411767

[64] Nurk S Walenz BP Rhie A Vollger MR Logsdon GA Grothe R HiCanu: accurate assembly of segmental duplications, satellites, and allelic variants from high-fidelity long reads. Genome Res (2020) 30(9):1291–305. 10.1101/gr.263566.120 32801147 PMC7545148

[65] Chaisson MJ Tesler G. Mapping single molecule sequencing reads using basic local alignment with successive refinement (BLASR): application and theory. BMC Bioinformatics (2012) 13:238. 10.1186/1471-2105-13-238 22988817 PMC3572422

[66] Chin CS Peluso P Sedlazeck FJ Nattestad M Concepcion GT Clum A Phased diploid genome assembly with single-molecule real-time sequencing. Nat Methods (2016) 13(12):1050–4. 10.1038/nmeth.4035 27749838 PMC5503144

[67] Mayakonda A Lin DC Assenov Y Plass C Koeffler HP. Maftools: efficient and comprehensive analysis of somatic variants in cancer. Genome Res (2018) 28(11):1747–56. 10.1101/gr.239244.118 30341162 PMC6211645

[68] Wickham H ggplot2: elegant graphics for data analysis. New York: Springer-Verlag (2016). Available from: https://ggplot2.tidyverse.org (Accessed 2009).

[69] Hunter JD. Matplotlib: a 2D graphics environment. Comput Sci Eng (2007) 9(3):90–5. 10.1109/MCSE.2007.55

[70] Bruce J Abeel T Shea T Priest M Abouelliel A Sakthikumar S Pilon: an integrated tool for comprehensive microbial variant detection and genome assembly improvement. PLoS ONE (2014) 9(11):e112963. 10.1371/journal.pone.0112963 25409509 PMC4237348

[71] Vaser R Sović I Nagarajan N Šikić M. Fast and accurate *de novo* genome assembly from long uncorrected reads. Genome Res (2017) 27(5):737–46. 10.1101/gr.214270.116 28100585 PMC5411768

[72] Yépez VA Gusic M Kopajtich R Mertes C Smith NH Alston CL Clinical implementation of RNA sequencing for Mendelian disease diagnostics. Genome Med (2022) 14(1):38. 10.1186/s13073-022-01019-9 35379322 PMC8981716

[73] Perešíni P Boža V Brejová B Vinař T. Nanopore base calling on the edge. Bioinformatics (2021) 37(24):4661–7. 10.1093/bioinformatics/btab528 34314502 PMC8665737

[74] Ledergerber C Dessimoz C. Base-calling for next-generation sequencing platforms. Brief Bioinform (2011) 12(5):489–97. 10.1093/bib/bbq077 21245079 PMC3178052

[75] Koboldt DC. Best practices for variant calling in clinical sequencing. Genome Med (2020) 12(91):91. 10.1186/s13073-020-00791-w 33106175 PMC7586657

[76] Schmieder R Edwards R. Quality control and preprocessing of metagenomic datasets. Bioinformatics (2011) 27(6):863–4. 10.1093/bioinformatics/btr026 21278185 PMC3051327

[77] Bolognini D Semeraro R Magi A. Versatile quality control methods for nanopore sequencing. Evol Bioinform Online (2019) 15:1176934319863068. 10.1177/1176934319863068 31384125 PMC6651670

[78] Frye M Harada BT Behm M He C. RNA modifications modulate gene expression during development. Science (2018) 361(6409):1346–9. 10.1126/science.aau1646 30262497 PMC6436390

[79] Field AE Robertson NA Wang T Havas A Ideker T Adams PD. DNA methylation clocks in aging: categories, causes, and consequences. Mol Cel (2018) 71(6):882–95. 10.1016/j.molcel.2018.08.008 PMC6520108 30241605

[80] Esteller M. Cancer epigenomics: DNA methylomes and histone-modification maps. Nat Rev Genet (2007) 8:286–98. 10.1038/nrg2005 17339880

[81] Kumar S Chinnusamy V Mohapatra T. Epigenetics of modified DNA bases: 5-methylcytosine and beyond. Front Genet (2018) 9(18):640. 10.3389/fgene.2018.00640 30619465 PMC6305559

[82] Duffy K Arangundy-Franklin S Holliger P. Modified nucleic acids: replication, evolution, and next-generation therapeutics. BMC Biol (2020) 18(112):112. 10.1186/s12915-020-00803-6 32878624 PMC7469316

[83] Kumar S Mohapatra T. Deciphering epitranscriptome: modification of mRNA bases provides a new perspective for post-transcriptional regulation of gene expression. Front Cel Dev. Biol. (2021) 9(16):628415. 10.3389/fcell.2021.628415 PMC8010680 33816473

[84] Soto J Rodriguez-Antolin C Vallespín E de Castro Carpeño J Ibanez de Caceres I. The impact of next-generation sequencing on the DNA methylation–based translational cancer research. Translational Res (2016) 169:1–18. 10.1016/j.trsl.2015.11.003 26687736

[85] Hirst M Marra MA. Next generation sequencing based approaches to epigenomics. Brief Funct Genomics (2010) 9(5-6):455–65. 10.1093/bfgp/elq035 21266347 PMC3080743

[86] Flusberg BA Webster DR Lee JH Travers KJ Olivares EC Clark TA Direct detection of DNA methylation during single-molecule, real-time sequencing. Nat Methods (2010) 7:461–5. 10.1038/nmeth.1459 20453866 PMC2879396

[87] Xu L Seki M. Recent advances in the detection of base modifications using the Nanopore sequencer. J Hum Genet (2020) 65:25–33. 10.1038/s10038-019-0679-0 31602005 PMC7087776

[88] Smolka M Paulin LF Grochowski CM Horner DW Mahmoud M Behera S Detection of mosaic and population-level structural variants with Sniffles2. Nat Biotechnol (2024). 10.1038/s41587-023-02024-y PMC11217151 38168980

[89] Stoiber M Quick J Egan R Lee JE Celniker S Neely RK De novo identification of DNA modifications enabled by genome-guided nanopore signal processing. bioRxiv 094672 (2017). 10.1101/094672

[90] Mandelker D Ceyhan-Birsoy O. Evolving significance of tumor-normal sequencing in cancer care. Trends Cancer (2020) 6(1):31–9. 10.1016/j.trecan.2019.11.006 31952779 PMC8923150

[91] Alkan C Coe B Eichler E. Genome structural variation discovery and genotyping. Nat Rev Genet (2011) 12:363–76. 10.1038/nrg2958 21358748 PMC4108431

[92] Zverinova S Guryev V. Variant calling: considerations, practices, and developments. Hum Mutat (2022) 43(8):976–85. 10.1002/humu.24311 34882898 PMC9545713

[93] Mahmoud M Gobet N Cruz-Dávalos DI Mounier N Dessimoz C Sedlazeck FJ. Structural variant calling: the long and the short of it. Genome Biol (2019) 20(246):246. 10.1186/s13059-019-1828-7 31747936 PMC6868818

[94] Mitsuhashi S Matsumoto N. Long-read sequencing for rare human genetic diseases. J Hum Genet (2020) 65:11–9. 10.1038/s10038-019-0671-8 31558760

[95] Schüle B McFarland KN Lee K Tsai YC Nguyen KD Sun C Parkinson’s disease associated with pure ATXN10 repeat expansion. Parkinson's Dis (2017) 3(27):27. 10.1038/s41531-017-0029-x PMC5585403 28890930

[96] McColgan P Tabrizi SJ. Huntington's disease: a clinical review. Eur J Neurol (2018) 25(1):24–34. 10.1111/ene.13413 28817209

[97] Sakamoto Y Zaha S Suzuki Y Seki M Suzuki A. Application of long-read sequencing to the detection of structural variants in human cancer genomes. Comput Struct Biotechnol J (2021) 19:4207–16. 10.1016/j.csbj.2021.07.030 34527193 PMC8350331

[98] Phillippy A. New advances in sequence assembly. Genome Res (2017) 27(5):xi–xiii. 10.1101/gr.223057.117 28461322 PMC5411783

[99] van Dijk EL Jaszczyszyn Y Naquin D Thermes C. The third revolution in sequencing technology. Trends Genet (2018) 34(9):666–81. 10.1016/j.tig.2018.05.008 29941292

[100] Nagarajan N Pop M. Sequence assembly demystified. Nat Rev Genet (2013) 14:157–67. 10.1038/nrg3367 23358380

[101] Chen Z Erickson DL Meng J. Benchmarking hybrid assembly approaches for genomic analyses of bacterial pathogens using Illumina and Oxford Nanopore sequencing. BMC Genomics (2020) 21:631. 10.1186/s12864-020-07041-8 32928108 PMC7490894

[102] Zhong Y Xu F Wu J Schubert J Li MM. Application of next generation sequencing in laboratory medicine. Ann Lab Med (2021) 41(1):25–43. 10.3343/alm.2021.41.1.25 32829577 PMC7443516

[103] Bartalucci N Romagnoli S Vannucchi AM. A blood drop through the pore: nanopore sequencing in hematology. Trends Genet (2022) 38(6):572–86. 10.1016/j.tig.2021.11.003 34906378

[104] Erlich RL Jia X Anderson S Banks E Gao X Carrington M Next-generation sequencing for HLA typing of class I loci. BMC Genomics (2011) 12:42. 10.1186/1471-2164-12-42 21244689 PMC3033818

[105] Klasberg S Surendranath V Lange V Schöfl G. Bioinformatics strategies, challenges, and opportunities for next generation sequencing-based HLA genotyping. Transfus Med Hemother (2019) 46(5):312–25. 10.1159/000502487 31832057 PMC6876610

[106] Garg S. Computational methods for chromosome-scale haplotype reconstruction. Genome Biol (2021) 22(1):101. 10.1186/s13059-021-02328-9 33845884 PMC8040228

[107] Cilibrasi R van Iersel L Kelk S Tromp J. The complexity of the single individual SNP haplotyping problem. Algorithmica (2007) 49:13–36. 10.1007/s00453-007-0029-z

[108] Sakamoto Y Sereewattanawoot S Suzuki A. A new era of long-read sequencing for cancer genomics. J Hum Genet (2020) 65:3–10. 10.1038/s10038-019-0658-5 31474751 PMC6892365

[109] Tarabichi M Demetter P Craciun L Maenhaut C Detours V. Thyroid cancer under the scope of emerging technologies. Mol Cel Endocrinol (2022) 541:111491. 10.1016/j.mce.2021.111491 34740746

[110] Muñoz-Barrera A Rubio-Rodríguez LA Díaz-de Usera A Jáspez D Lorenzo-Salazar JM González-Montelongo R From samples to germline and somatic sequence variation: a focus on next-generation sequencing in melanoma research. Life (Basel) (2022) 12(11):1939. 10.3390/life12111939 36431075 PMC9695713

[111] Vollmers AC. Long noncoding RNA. Introduction and overview. In: Crusio WE Dong H Radeke HH Rezaei N Steinlein O Xiao J, editors. Advances in experimental medicine and biology. Springer (2022). 10.1007/978-3-030-92034-0_1 35220562

[112] Park EG Pyo SJ Cui Y Yoon SH Nam JW. Tumor immune microenvironment lncRNAs. Brief Bioinform (2022) 23(1):bbab504. 10.1093/bib/bbab504 34891154 PMC8769899

[113] Li X Wang C-Y. From bulk, single-cell to spatial RNA sequencing. Int J Oral Sci (2021) 13(36):36. 10.1038/s41368-021-00146-0 34782601 PMC8593179

[114] Depledge DP Srinivas KP Sadaoka T Bready D Mori Y Placantonakis DG Direct RNA sequencing on nanopore arrays redefines the transcriptional complexity of a viral pathogen. Nat Commun (2019) 10:754. 10.1038/s41467-019-08734-9 30765700 PMC6376126

[115] Jovic D Liang X Zeng H Lin L Xu F Luo Y. Single-cell RNA sequencing technologies and applications: a brief overview. Clin Trans Med (2022) 12(3):e694. 10.1002/ctm2.694 PMC8964935 35352511

[116] Razavi P Li BT Brown DN Jung B Hubbell E Shen R High-intensity sequencing reveals the sources of plasma circulating cell-free DNA variants. Nat Med (2019) 25:1928–37. 10.1038/s41591-019-0652-7 31768066 PMC7061455

[117] Song P Wu LR Yan YH Zhang JX Chu T Kwong LN Limitations and opportunities of technologies for the analysis of cell-free DNA in cancer diagnostics. Nat Biomed Eng (2022) 6(3):232–45. 10.1038/s41551-021-00837-3 35102279 PMC9336539

[118] Shieh JTC. Genomic technologies to improve variation identification in undiagnosed diseases. Ped Neonatal (2023) 64(S1):S18–S21. 10.1016/J.pedneo.2022.10.002 36428199

[119] Macken WL Vandrovcova J Hanna MG Pitceathly RDS. Applying genomic and transcriptomic advances to mitochondrial medicine. Nat Rev Neurol (2021) 17:215–30. 10.1038/s41582-021-00455-2 33623159

[120] Esteller M. Epigenetic gene silencing in cancer: the DNA hypermethylome. Hum Mol Genet (2007) 16(Spec No 1):R50–9. 10.1093/hmg/ddm018 17613547

[121] Lakshminarasimhan R Liang G. The role of DNA methylation in cancer. Adv Exp Med Biol (2016) 945:151–72. 10.1007/978-3-319-43624-1_7 27826838 PMC7409375

[122] Abante J Kambhampati S Feinberg AP Goutsias J. Estimating DNA methylation potential energy landscapes from nanopore sequencing data. Sci Rep (2021) 11(1):21619. 10.1038/s41598-021-00781-x 34732768 PMC8566571

[123] Jhanwar S Deogade A. 5-Methylcytosine and 5-hydroxymethylcytosine signatures underlying pediatric cancers. Epigenomes (2019) 3(2):9. 10.3390/epigenomes3020009 34968232 PMC8594703

[124] Zhou D Guo S Wang Y Zhao J Liu H Zhou F Functional characteristics of DNA N6-methyladenine modification based on long-read sequencing in pancreatic cancer. Brief Funct Genomics (2023) 23:150–62. 10.1093/bfgp/elad021 37279592

[125] Brockley LJ Souza VGP Forder A Pewarchuk ME Erkan M Telkar N Sequence-based platforms for discovering biomarkers in liquid biopsy of non-small-cell lung cancer. Cancers (Basel) (2023) 15(8):2275. 10.3390/cancers15082275 37190212 PMC10136462

[126] Ibrahim J Peeters M Van Camp G Op de Beeck K. Methylation biomarkers for early cancer detection and diagnosis: current and future perspectives. Eur J Cancer (2023) 178:91–113. 10.1016/j.ejca.2022.10.015 36427394

[127] Sahm F Schrimpf D Jones DTW Meyer J Kratz A Reuss D Next-generation sequencing in routine brain tumor diagnostics enables an integrated diagnosis and identifies actionable targets. Acta Neuropathol (2016) 131(6):903–10. 10.1007/s00401-015-1519-8 26671409

[128] Arts P Simons A AlZahrani MS Yilmaz E AlIdrissi E van Aerde KJ Exome sequencing in routine diagnostics: a generic test for 254 patients with primary immunodeficiencies. Genome Med (2019) 11:38. 10.1186/s13073-019-0649-3 31203817 PMC6572765

[129] Breinholt MF Nielsen K Schejbel L Fassi DE Schöllkopf C Novotny GW The value of next-generation sequencing in routine diagnostics and management of patients with cytopenia. Int J Lab Hematol (2022) 44(3):531–7. 10.1111/ijlh.13802 35142436

[130] Fogel BL Lee H Strom SP Deignan JL Nelson SF. Clinical exome sequencing in neurogenetic and neuropsychiatric disorders. Ann N Y Acad Sci (2016) 1366(1):49–60. 10.1111/nyas.12850 26250888 PMC4744590

[131] Schmidt J Blessing F Fimpler L Wenzel F. Nanopore sequencing in a clinical routine laboratory: challenges and opportunities. Clin Lab (2020) 66(6). 10.7754/Clin.Lab.2019.191114 32538066

[132] Olivucci G Iovino E Innella G Turchetti D Pippucci T Magini P. Long read sequencing on its way to the routine diagnostics of genetic diseases. Front Genet (2024) 15:1374860. 10.3389/fgene.2024.1374860 38510277 PMC10951082

[133] Eagle SHC Robertson J Bastedo DP Liu K Nash JHE. Evaluation of five commercial DNA extraction kits using Salmonella as a model for implementation of rapid Nanopore sequencing in routine diagnostic laboratories. Access Microbiol (2023) 5(2):000468v3. 10.1099/acmi.0.000468.v3 PMC9996181 36910509

[134] Erdmann H Schöberl F Giurgiu M Leal Silva RM Scholz V Scharf F Parallel in-depth analysis of repeat expansions in ataxia patients by long-read sequencing. Brain (2023) 146(5):1831–43. 10.1093/brain/awac377 36227727

[135] Matern BM Olieslagers TI Groeneweg M Duygu B Wieten L Tilanus MGJ Long-read nanopore sequencing validated for human leukocyte antigen class I typing in routine diagnostics. J Mol Diagn (2020) 22(7):912–9. 10.1016/j.jmoldx.2020.04.001 32302780

[136] Buenestado-Serrano S Herranz M Otero-Sobrino Á Molero-Salinas A Rodríguez-Grande C Sanz-Pérez A Accelerating SARS-CoV-2 genomic surveillance in a routine clinical setting with nanopore sequencing. Int J Med Microbiol (2024) 314:151599. 10.1016/j.ijmm.2024.151599 38290400

